# Oral nimodipine treatment has no effect on amyloid pathology or neuritic dystrophy in the 5XFAD mouse model of amyloidosis

**DOI:** 10.1371/journal.pone.0263332

**Published:** 2022-02-02

**Authors:** Katherine R. Sadleir, Jelena Popovic, Ammaarah Khatri, Robert Vassar

**Affiliations:** Department of Neurology, Feinberg School of Medicine, Northwestern University, Chicago, IL, United States of America; National Center of Neurology and Psychiatry (NCNP), JAPAN

## Abstract

Dysregulation of calcium homeostasis has been hypothesized to play a role in Alzheimer’s disease (AD) pathogenesis. Increased calcium levels can impair axonal transport, disrupt synaptic transmission, and ultimately lead to cell death. Given the potential role of calcium dyshomeostasis in AD, there is interest in testing the ability of already approved drugs targeting various calcium channels to affect amyloid pathology and other aspects of disease. The objective of this study was to test the effects of FDA-approved L-type calcium channel antagonist nimodipine on amyloid accumulation and dystrophic neurite formation in 5XFAD mice, a mouse model of amyloid pathology. 5XFAD transgenic mice and non-transgenic littermates were treated with vehicle or nimodipine-containing chow from two to eight months of age, then brains were harvested and amyloid pathology assessed by immunoblot and immunofluorescence microscopy analyses. Nimodipine was well tolerated and crossed the blood brain barrier, as expected, but there was no effect on Aβ accumulation or on the relative amount of neuritic dystrophy, as assessed by either immunoblot, dot blot or immunofluorescence imaging of Aβ42 and dystrophic neurite marker LAMP1. While we conclude that nimodipine treatment is not likely to improve amyloid pathology or decrease neuritic dystrophy in AD, it is worth noting that nimodipine did not worsen the phenotype suggesting its use is safe in AD patients.

## Introduction

Alzheimer’s disease (AD) is the most common neurodegenerative disease of the elderly and causes irreversible memory loss and behavioral changes, ultimately leading to death [1,2], and, with the possible exception of aducanumab/Aduhelm, there are no disease modifying therapeutics available. The two main pathological hallmarks of AD are extracellular amyloid plaques, composed of aggregated Aβ peptides, and intracellular neurofibrillary tangles composed of hyperphosphorylated tau [1,2]. Aβ peptides are generated by the sequential cleavages of the amyloid precursor protein (APP) by the β-secretase, BACE1 [3–6], followed by γ-secretase. The amyloid hypothesis posits that the early accumulation of Aβ leads to downstream tau pathology, neuroinflammation, loss of synapses and neurons, and memory impairments [7]. Despite the wealth of data supporting this hypothesis, so far, clinical trials of drugs targeting amyloid generation have failed to demonstrate unequivocal efficacy, leading to interest in targeting other potential pathways that contribute to AD pathology and memory loss.

The calcium hypothesis, which was first formalized in 1976 [8] and updated in 2017 [9] complements the amyloid hypothesis. In this view, the pathologies and risk factors of AD, through different mechanisms, all converge on calcium dysregulation leading to synaptic and neuronal dysfunction, and cell death. There are multiple ways in which amyloid pathology can lead to calcium dysregulation, such as disrupting the plasma membrane [10], by activating NMDA receptors [11], and by interacting with calcium channels [12]. Though the mechanism is not yet determined, and indeed multiple mechanisms may be contributing, there is a large amount of evidence for calcium dyshomeostasis in the immediate proximity of amyloid plaques [13–15]. In addition to elevated calcium [15], there is loss of mitochondria [16] and synapses, astrocyte and microglia activation and dystrophic neurites, all pointing to toxicity of accumulated amyloid. We hypothesize that local calcium elevation near plaques disrupts microtubules and leads to impaired protein transport and degradation causing the accumulation of synaptic and axonal proteins in swollen dystrophies around the plaque, which in turn contribute to increased Aβ deposition [17]. While we favor this hypothesis, there are other mechanisms that can lead to calcium dysregulation associated with AD, such as alterations to the calcium ER leak function of presenilin which are associated with familial AD presenilin mutations [18,19]. One way to ameliorate the halo of toxicity around the plaque might be to block calcium elevation with calcium channel blockers, many of which are already FDA approved.

Epidemiological studies indicate that there may be protective effects of calcium channel blockers in slowing [20,21] or preventing AD [22]. The dihydropyridines (DHP) are compounds that selectively antagonize the L-type voltage gated calcium channels expressed mainly on neuronal cell bodies and dendrites [23–25]. The DHP nimodipine effectively crosses the blood brain barrier [26] and is effective in the prevention of ischemia after subarachnoid hemorrhage (reviewed in [27]), likely through vasodilation. There are data indicating nimodipine is effective in preserving cognitive function in people with AD and other dementia subtypes [28,29], perhaps by increasing blood flow or decreasing calcium influx into neurons. Nimodipine also decreased spontaneous firing and reduced intracellular calcium concentration in arcuate neuropeptide Y neurons of an amyloid pathology mouse model [30]. Given these various data suggesting the beneficial effects of nimodipine in AD, we treated 5XFAD mice with oral nimodipine during the active phases of plaque deposition and growth and quantified amyloid plaques and neuritic dystrophies to directly investigate the effects of nimodipine on pathology in a mouse model of amyloidosis in order to better inform preclinical studies of the use of this drug for the treatment of AD.

## Materials and methods

### Mice

**Fig 1:**
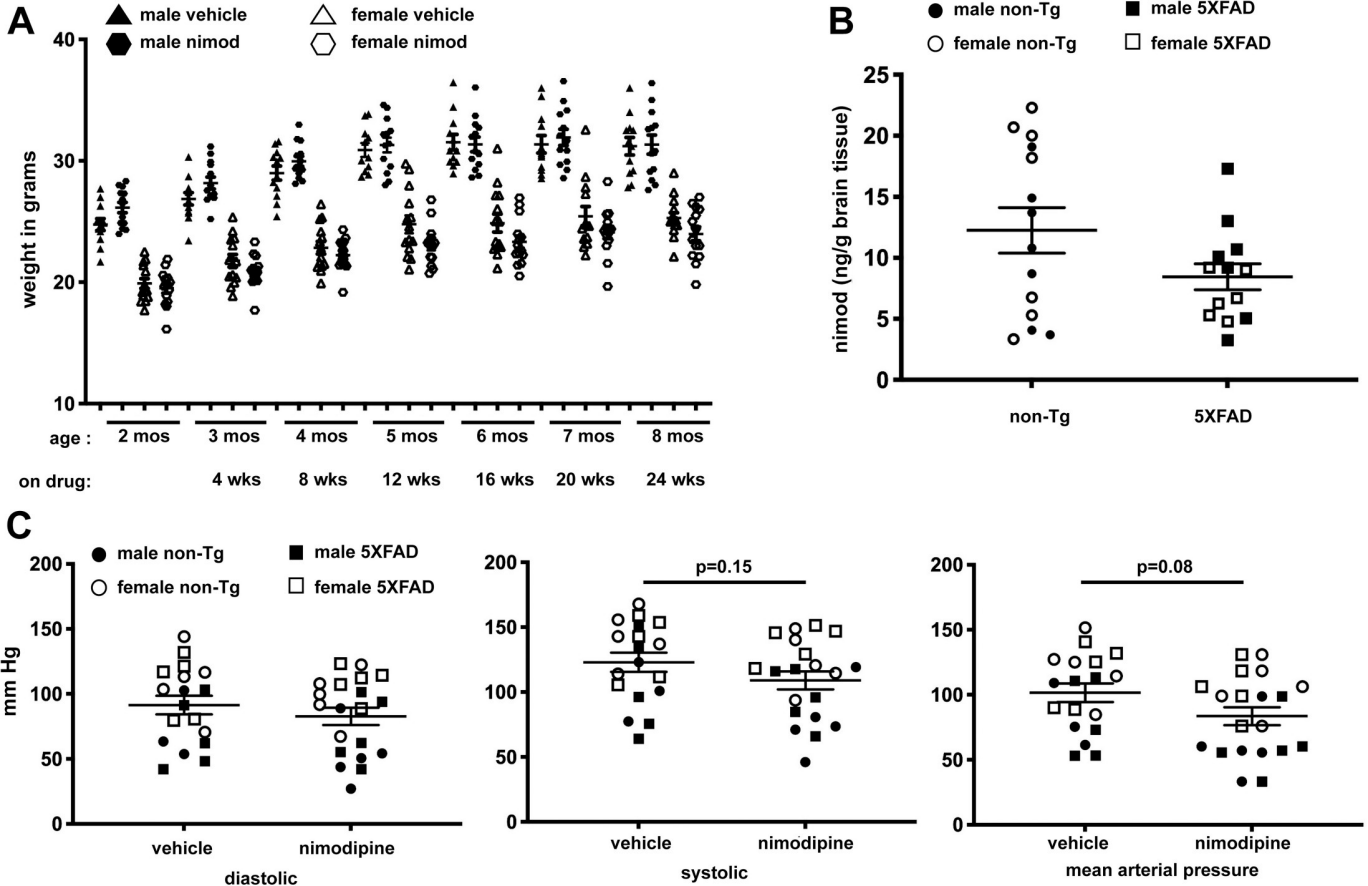
Oral nimodipine accumulates in the brain and has minimal effect on blood pressure. (A) 5XFAD and non-transgenic control mice were weighed at 2 months and started on nimodipine diet, then weighed monthly until brain harvest at 8 months of age. Nimodipine treatment did not affect weight gain or maintenance in either males or females. (B) Nimodipine concentration in the brains of nimodipine-treated mice were determined by mass spectrometry. While there was notable animal to animal variation, there was no significant difference in nimodipine concentration between the genotypes or sexes. (C) At 8 months of age, mice were subjected to blood pressure measurement using a tail cuff system. We observed a non-significant trend for reduced blood pressure in nimodipine-treated mice.

5XFAD mice were bred in house by crossing 5XFAD transgenic males to B6/SJL F1 hybrid females and genotyped as described [31]. Nimodipine was purchased from R&D systems (product #0600) and incorporated into mouse chow at 300 ppm by Envigo. Mice were fed either nimodipine or vehicle chow starting at 2.5 months, then harvested at 8 months. Animals were weighed at the beginning of the experiment and every 4 weeks thereafter and demonstrated that nimodipine had no effect on weight gain (Fig 1A). The food was also weighed weekly to assess consumption. Based on the weight of food consumed per cage and the average weight of the mice, females received doses ranging from 40–51 mg/kg/day, while males received 33–41 mg/kg/day. Animal numbers were as follows: Vehicle non-transgenic: 7 female, 5 male; vehicle 5XFAD: 7 female, 7 male, nimodipine non-transgenic: 7 female, 7 male, nimodipine 5XFAD; 7 female, 6 male. Two weeks before harvest (approximately 7.5 months of age) the mice underwent behavioral testing on fear conditioning and Y-maze, as described below. The week before harvest, blood pressure was measured using a CODA High Throughput System Noninvasive Blood Pressure System (Kent Scientific). Mice were sacrificed by a lethal dose of ketamine/xylazine, followed by transcardial perfusion with 10 ml ice cold 1xPBS containing protease and phosphatase inhibitors. One hemibrain was dissected into hippocampus, cortex and cerebellum which were snap-frozen separately. The other hemibrain was drop fixed in 10% buffered formalin overnight at 4°C, then transferred to 20% w/v sucrose in 1xPBS for 24 hours, then stored in 30% w/v sucrose in 1xPBS with azide. All animal work was done with the approval of the Northwestern University IACUC, assurance number A3283-01.

### Quantification of nimodipine in brain

Frozen cerebellar tissues from nimodipine-treated and vehicle-treated mice were shipped to Absorption Systems CRO (Exton, PA) along with a sample of nimodipine for generation of a standard curve. Tissue was processed by Absorption Systems for nimodipine measurement by liquid chromatography tandem mass spectrometry (LC-MS/MS). To create the standard curve, nimodipine was added in known concentrations to brain lysate from a mouse that did not consume nimodipine-containing chow. These samples, along with the tissue from mice that consumed chow with or without nimodipine were analyzed by LC-MS/MS, and nimodipine concentration in brain tissue was determined by comparison to the standard curve.

### Immunoblotting

Snap frozen cortices and hippocampi were homogenized in 1000μl or 250μl, respectively, of T-PER Tissue Protein Extraction Reagent (ThermoFisher), supplemented with protease inhibitors (Calbiochem) and Halt Phosphatase Inhibitor Cocktail (Thermo Scientific). Protein concentration was quantified using BCA Assay (Pierce). 20ug of protein was boiled 10 min in 1x LDS sample loading buffer, then separated on 4–12% NuPage Bis-Tris Bolt gels (FisherScientific) in MES buffer (FisherScientific). Protein was transferred overnight to PVDF (Millipore), stained with 0.1% Ponceau in 5% w/v acetic acid and imaged. Membrane was rinsed well, blocked, then probed with the following primary antibodies: anti-APP antibody (6E10, BioLegend 803001, 1:2000), anti-BACE1 (3D5 [32], 1:1000), anti-LAMP1 (Cell Signaling rabbit mAb #3243, 1:3000), anti-LC3B (Cell Signaling rabbit mAb #3868 1:4000), anti-β-tubulin (TuJ1, gift of Dr. Nicholas Kanaan, 1:10,000) followed by HRP-conjugated anti-mouse or anti-rabbit secondary antibody (Vector Laboratories 1:10,000). 5% milk was used as a blocking agent. Blots were visualized using chemiluminescence (ECL+ or SuperSignal West Pico Plus, ThermoFisher), band intensities measured using a FluorChemR imager (ProteinSimple), then quantified with Alphaview software (ProteinSimple). Signal intensities were normalized to that of tubulin. Statistical analyses were performed as described below. For these analyses, multiple gels were cut into horizontal strips and stacked so all samples for a given protein were transferred to a single piece of PVDF membrane. Putting all samples on one membrane eliminated the need to account for variation in transfer, antibody incubation, and ECL application that can occur between blots.

### Dot blotting

For Aβ42 dot blots, 7.8 μl of 5 mg/ml brain homogenates were added to 12.2 μl of freshly made 8.2 M guanidine hydrochloride (GuHCl); 82 mM Tris HCl (pH 8.0) (5 M GuHCl final) and mixed for three days on a nutator. 1μl of this solution, containing 1.95 μg total protein in 5M GuHCl was pipetted in triplicate on a gridded nitrocellulose membrane, dried for one hour at 37°C, washed in TBS-T then water, and stained with Ponceau S. Four identical blots were made and incubated in either 1:4000 anti-Aβ42 rabbit monoclonal antibody (clone H31L21, Invitrogen, #700254), 1:4000 anti-Aβ total mouse monoclonal (3D6, Elan), or 5% milk only (primary delete), followed by HRP-conjugated secondary goat anti-rabbit or horse anti-mouse antibodies (Vector Labs, PI-1000, PI-2000). The blots incubated with anti-Aβ antibody or primary delete were developed together with ECL Plus (Peirce) and imaged simultaneously using a FluorChemR imager (ProteinSimple), then quantified with Alphaview software (ProteinSimple). The secondary and primary antibody delete controls indicated no IgG background. Signal intensities were normalized to Ponceau S staining, triplicates averaged, and statistical analyses were performed as described below.

### Immunofluorescence and microscopy

10% formalin-fixed hemibrains were sectioned at 30μm on a freezing sliding microtome and collected in cryopreserve (30% w/v sucrose, 30% ethylene glycol in 1X PBS). Three male and three female mice from each treatment group were selected at random for immunofluorescent staining. Three sections between Bregma -1.58mm to Bregma -2.54mm were stained and imaged per mouse. The primary antibodies used were 1:3000 rat moncolonal anti-LAMP1 (clone 1D4B, DSHB) and 1:3000 rabbit monoclonal anti-Aβ42 antibody (clone H31L21, Invitrogen, #700254) in 1% BSA TBS-T at 4°C overnight. Following washes, sections were incubated for 2 hours at room temperature with the following secondary antibodies and stains: 1:3000 donkey anti-rat Alexa 488 (ThermoFisher Scientific), donkey anti-rabbit 647 (ThermoFisher Scientific), 300 nM DAPI, and 1:30,000 dilution of 1 mg/ml Thiazine Red. Sections were mounted with Prolong Gold (Molecular Probes) and images acquired on a Nikon Ti2 Eclipse widefield microscope with a 10x objective, using NIS Elements software high content method to capture and tile whole sections, or Nikon A1R confocal with a 60x objective. All image acquisition settings were maintained the same between treatment groups and genotypes. For image analysis, regions of interest (ROIs) were drawn in cortex and hippocampus. Using the General Analysis tool, thresholding was set to distinguish Aβ42, Thiazine Red, and LAMP1 positive regions, then the percent area covered by each stain from the hippocampal or cortical ROI was calculated. Using the same sections, thresholding in the General Analysis tool was used to define sub-ROIs that correspond to individual plaques having both Aβ42 and LAMP1 positive pixels within cortical and hippocampal ROIs. The General Analysis tool was used to measure area covered by Aβ42 and by LAMP1 in a given sub-ROI (plaque) and the ratio between LAMP1:Aβ42 was calculated in Excel; 516–2237 plaques were analyzed per mouse in cortex and 364–1467 plaques in hippocampus. Six mice (3 females and 3 males) per genotype/treatment group were analyzed, using 3 sections per mouse. In the cortex, 67–248 plaques in the in the 50–200 μm^2^ size range were analyzed per mouse, 317–988 in the 200–450 μm^2^ range, 100–703 in the 450–800 μm^2^ range, 21–236 in the 800–1250 μm^2^ range, and 1–40 in the 1250–1800 μm^2^ range. In the hippocampus, 57–202 plaques in the 50–200 μm^2^ size range were analyzed per mouse, 206–670 in the 200–450 μm^2^ range, 77–387 in the 450–800 μm^2^ range, 11–137 in the 800–1250 μm^2^ range, and 0–46 in the 1250–1800 μm^2^ range.

### Statistics

Student’s two-tailed t-test and ANOVA were done using InStat software (GraphPad Software, Inc., San Diego, CA) to compare means of the various genotypes, genders, and treatment groups. * 0.05 > p > 0.01 ** 0.01 > p > 0.001 *** 0.001 > p > 0.0001; Error bars = S.E.M.

## Results

### Nimodipine is well-tolerated and crosses the blood-brain barrier

Male and female 5XFAD mice and non-transgenic littermates were fed chow containing 300 ppm nimodipine starting at 9 weeks of age, which corresponds with the early stage of amyloid deposition [31]. Animals were weighed at the beginning of the experiment and every 4 weeks thereafter and it was demonstrated that nimodipine had no effect on weight gain (Fig 1A). The food was also weighed weekly to assess consumption. Based on the weight of food consumed per cage and the average weight of the mice, females received doses ranging from 40–51 mg/kg/day, while males received 33–41 mg/kg/day.

At 8 months of age, y-maze and fear conditioning behavioral tests were performed to test for effects on learning and memory, but no significant differences were found between non-Tg and 5XFAD on Y-maze or fear conditioning, limiting our ability to draw conclusions about the effects of nimodipine on cognition in the context of amyloid pathology (S1 Fig).

After completing behavioral testing, mice underwent blood pressure measurement using a tail cuff system. We observed that nimodipine treated mice had a non-significant trend for reduced blood mean arterial pressure (p = 0.08) (Fig 1C), which is consistent with the known effects nimodipine as a vasodilator, and reports of decreased blood pressure in humans [33]. When groups were broken down by sex, the nimodipine-related blood pressure reduction trend remained, but still was not significant in either male or female cohort. There was a significant sex difference between males and females within both the vehicle and nimodipine-treated groups, with females having significantly elevated systolic, diastolic and mean arterial pressure compared to males (Fig 1C). While sex differences in blood pressure have been reported, generally male mice have higher blood pressure than females, rather than lower as observed here [34,35]. No blood pressure differences were observed between non-transgenic and 5XFAD mice with either vehicle or nimodipine treatment.

After transcardial perfusion and tissue harvest, the cerebellum of each nimodipine-treated mouse was sent for analysis by mass spectrometry to measure levels of drug in the brain. We observed a wide range of nimodipine concentrations from 3.3–22.3 ng/gm brain tissue, but no significant differences between non-Tg and 5XFAD, or between males and females (Fig 1B). All were above the lowest value of the standard curve, and all blank (vehicle) brain samples had no signal, confirming the specificity of the assay.

### Nimodipine does not reduce BACE1 or LAMP1 accumulation in 5XFAD mice

**Fig 2:**
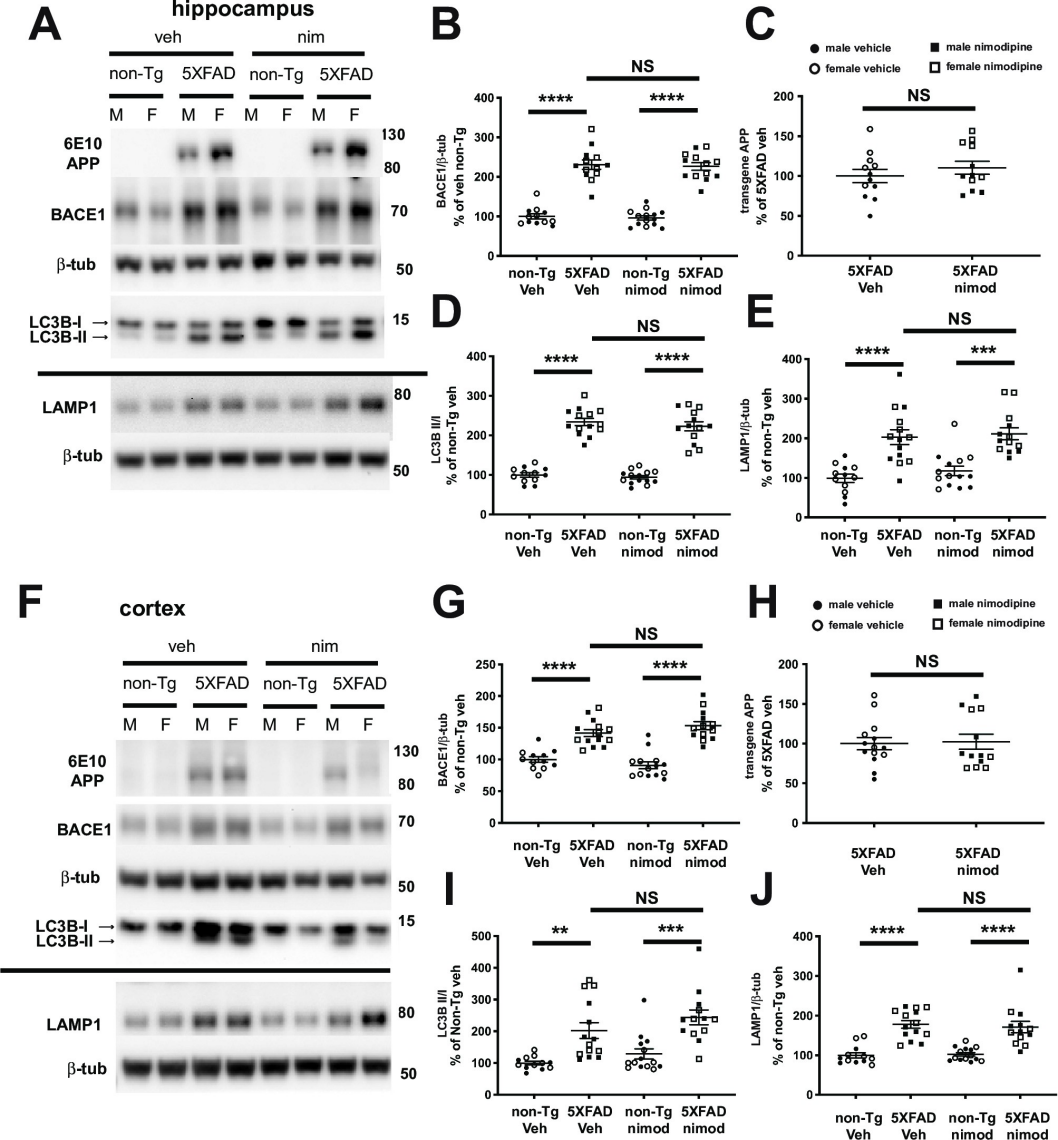
Nimodipine does not decrease markers of neuritic dystrophy in 5XFAD transgenic mice. **(**A and F) Representative immunoblots of hippocampal (A) and cortical (F) lysates of non-transgenic and 5XFAD mice treated with vehicle or nimodipine. (B-E and G-J) Quantification of BACE1 (B, G), transgenic APP (C, H), LAMP1 (E, J) (all normalized to β-tubulin) and LC3B-II/ LC3B-I (D, I). As expected, BACE1, LAMP1 and LC3B-II/ LC3B-I were elevated in vehicle-treated 5XFAD compared to vehicle non-transgenic mice. Similarly, BACE1, LAMP1 and LC3B-II/ LC3B-I were also increased in nimodipine-treated 5XFAD compared to nimodipine treated non-transgenic mice, indicating that nimodipine did not decrease the accumulation of these dystrophic neurite marker proteins. Nimodipine had no effect on expression of transgenic human APP. F, female; M, male; * 0.05 > p > 0.01 ** 0.01 > p > 0.001 *** 0.001 > p > 0.0001; **** 0.0001 > p; Error bars = S.E.M.

BACE1 and LAMP1 greatly accumulate in dystrophic neurites that surround amyloid plaques [17,32,36–38], and therefore their increases in the brain largely reflect the amount of neuritic dystrophy in 5XFAD brains. To assess the effect of nimodipine on BACE1 and LAMP1 accumulation, these proteins were measured by immunoblotting of homogenates from cortex and hippocampus of nimodipine- and vehicle-treated 5XFAD mice (Fig 2). As expected from previous studies [32,39,40], BACE1 was significantly elevated in 5XFAD compared to non-Tg mice in both cortex and hippocampus. However, we observed no significant difference between vehicle and nimodipine-treated 5XFAD mice, indicating no effect of the drug on BACE1 or LAMP1 accumulation (Fig 2B and 2D). The lysosome/early endosome protein LAMP1 has also been shown to accumulate in dystrophic neurites around plaques [36], and like BACE1 was significantly elevated in 5XFAD compared to non-Tg mice, but showed no difference between 5XFAD treated with vehicle or nimodipine (Fig 2B and 2D). Dystrophic neurites also accumulate autophagic intermediates, which is reflected in an increased ratio of LC3B II/I [37]. To determine if nimodipine had any effect on autophagy in dystrophic neurites, the ratio of phosphatidylethanolamine-conjugated LC3B II to LC3B I was quantified by immunoblot. As previously observed, LC3B II/I ratio was significantly elevated in 5XFAD compared to non-transgenic littermates, but we found no difference between vehicle and nimodipine-treated 5XFAD mice, indicating that nimodipine had no effect on autophagic marker buildup in dystrophies (Fig 2B and 2D). Finally, the genotypes of all the mice were verified using human Aβ-specific antibody 6E10, and it was confirmed that nimodipine had no effect on transgenic expression of APP protein.

### Nimodipine does not affect levels of amyloid in 5XFAD mice

**Fig 3:**
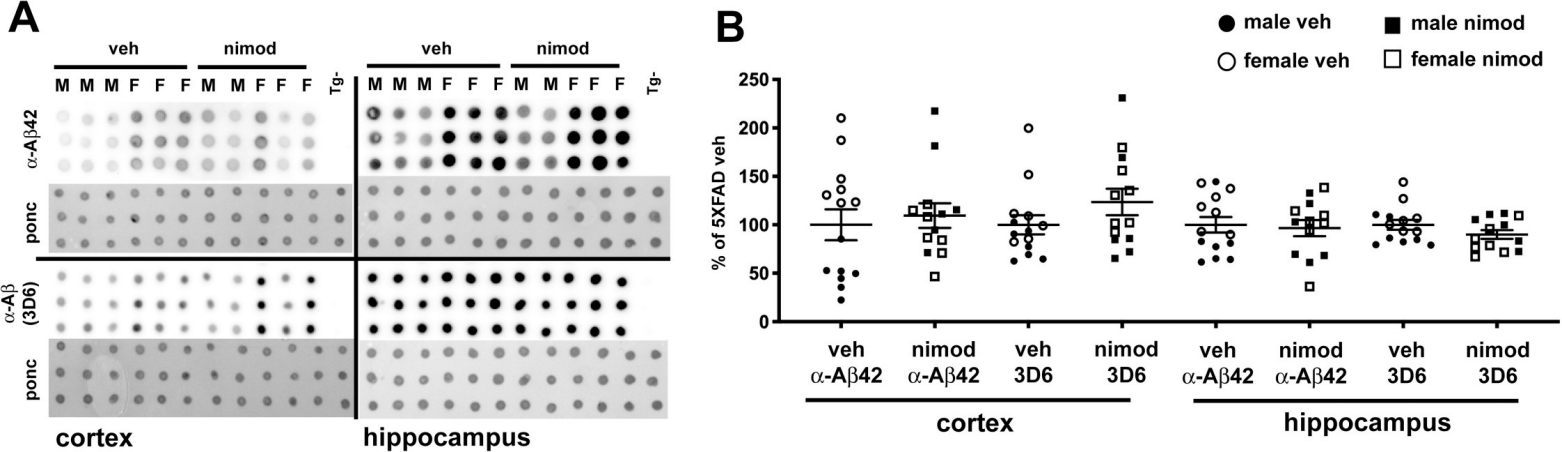
Nimodipine does not affect Aβ levels in cortex or hippocampus. (A) Representative dot blots of Aβ42 and Aβ1-x in cortex and hippocampus homogenates from 5XFAD mice treated with either vehicle or nimodipine. Non-transgenic samples were included to demonstrate the assay’s specificity for transgene-encoded Aβ. (B) Quantification of Aβ42 and Aβ (with 3D6 antibody). We observed no significant difference for either Aβ species between vehicle and nimodipine-treated mice in either cortex or hippocampus. We saw a trend for females to have higher levels of Aβ, as expected from previous studies.

To assess amyloid levels, we measured Aβ42 and total Aβ by dot blot (Fig 3). While a dot blot does not provide the same sensitivity or absolute quantification of and ELISA test, it is a efficient and cost-effective way to measure relative levels of Aβ species in the brain. Several previous studies have demonstrated the validity of this method [17,39–42] and it correlates well with ELISA data [39]. Nimodipine had no effect on Aβ42 or total Aβ levels in either cortex or hippocampus. As we have previously observed [39], we saw a trend for females to have higher Aβ levels than males on average, but this was not statistically significant.

**Fig 4:**
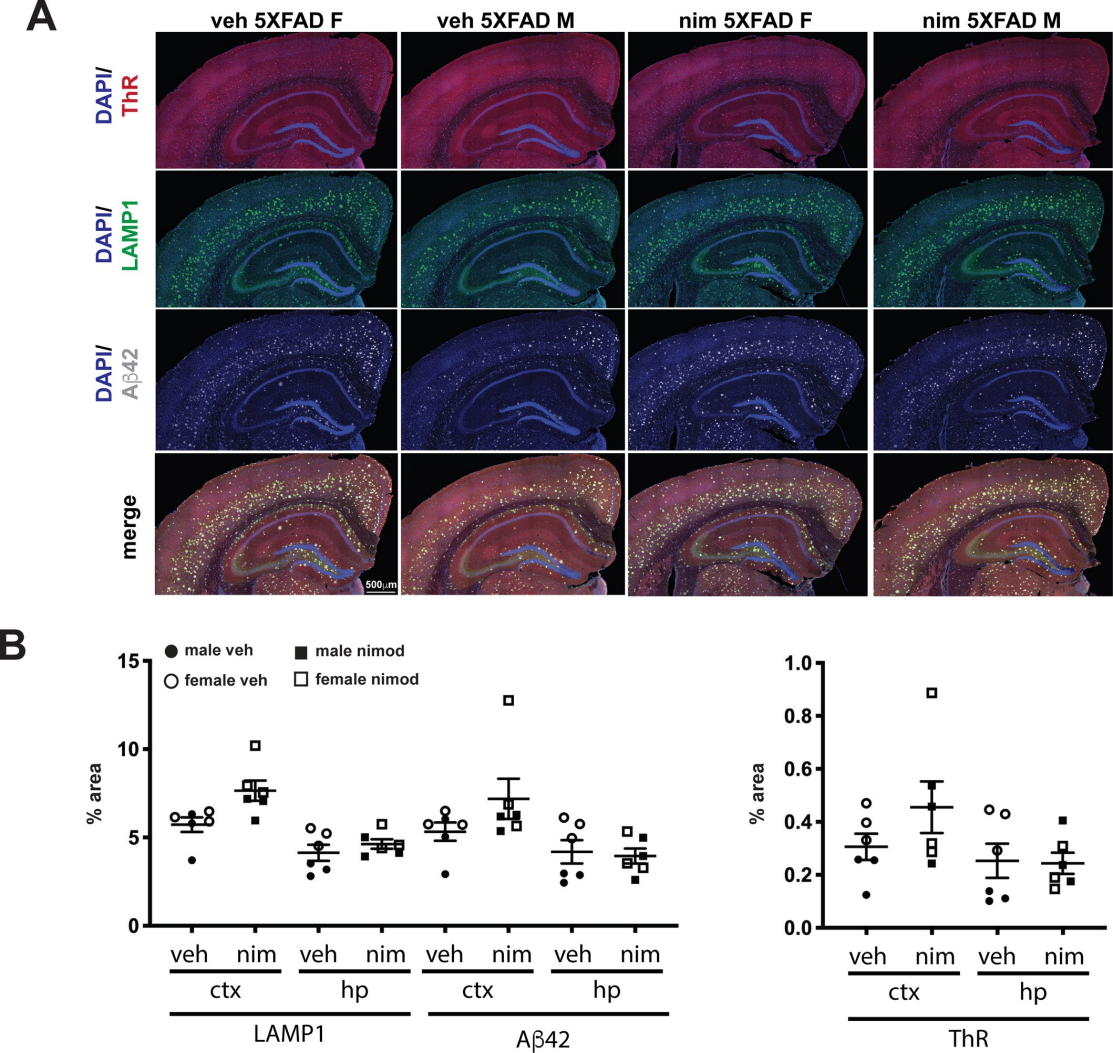
Nimodipine does not affect areas of amyloid plaques and dystrophic neurites in cortex or hippocampus. (A) Representative sections stained with Thiazine Red (red) for dense core plaques, anti-LAMP1 antibody (green) for dystrophic neurites, and anti- Aβ42 (white) for all plaques and DAPI (blue) nuclei. B and C) Quantification of the percent area covered by LAMP1, Aβ42 (B) or Thiazine Red (C) staining. Regions of interest were hand-drawn in cortex and hippocampus using Nikon NIS Elements software, and the percent area covered by each stain was calculated. There was no significant difference in LAMP1, Aβ42 or Thiazine Red staining between the brains of vehicle and nimodipine-treated mice. We observed a trend for females to have higher levels of Aβ42 and Thiazine Red, but was not statistically significant. ctx, cortex; hp, hippocampus; ThR, thiazine red. Error bars = SEM. Scale bar = 500 μm.

To confirm our biochemical measures of cerebral Aβ accumulation, we performed Aβ immunofluorescence microscopy and image analysis on three mice selected at random from each group (Fig 4). We used two amyloid stains, Thiazine red that is selective for fibrillar cores of Aβ plaques and anti-Aβ42 antibody that recognizes Aβ42 regardless of its aggregation state. In addition, we used immunostaining with an anti-LAMP1 antibody as a marker of dystrophic neurites. We quantified and averaged three sections per animal and determined the percent area in the cortex and hippocampus that was positive for Aβ42, LAMP1 and thaizine red. We found no significant difference between vehicle and nimodipine treated animals for any of the measures, confirming that plaque deposition is not affected by nimodipine.

### Nimodipine does not affect the relative amounts of LAMP1 and Aβ42

**Fig 5:**
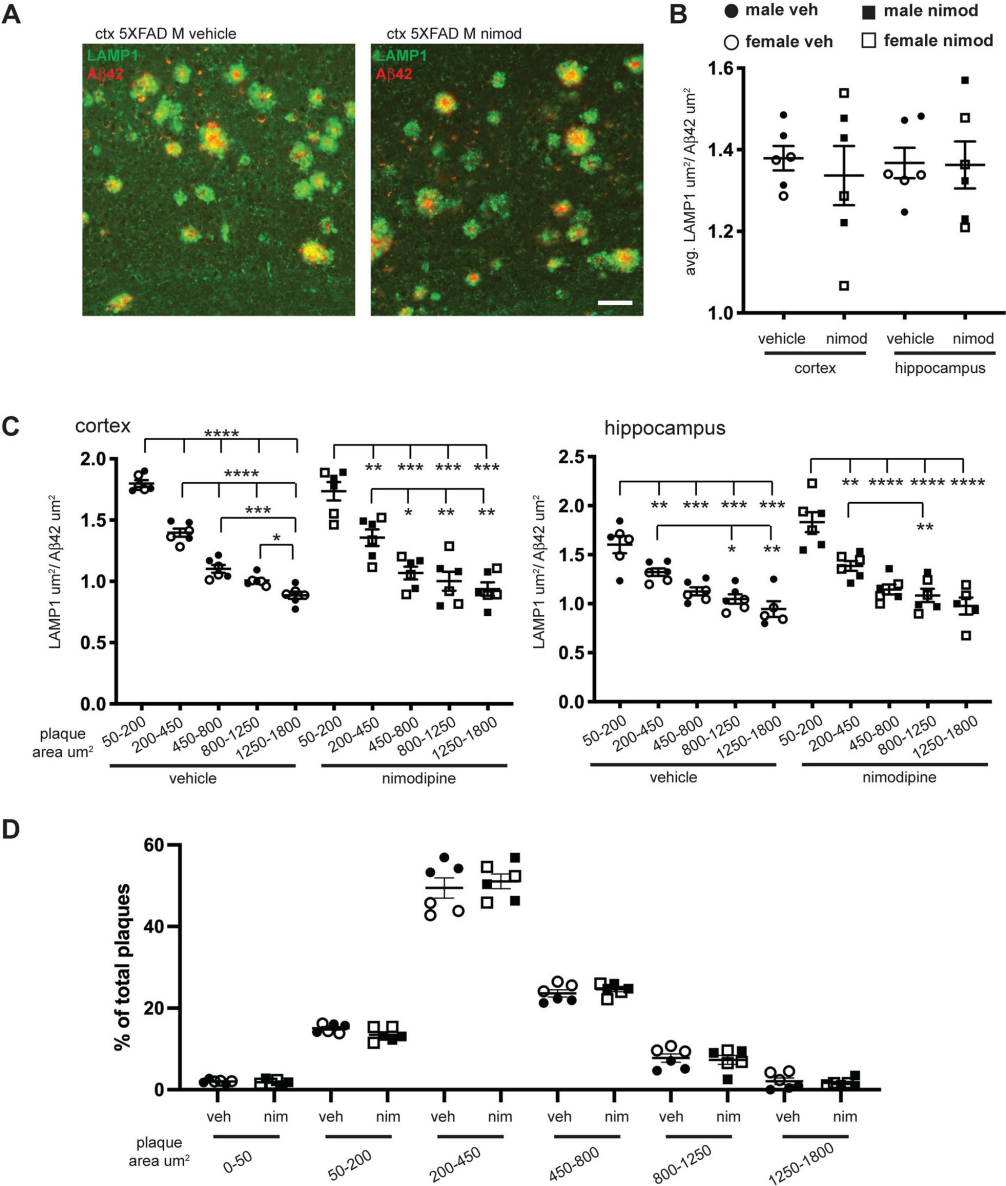
Dystrophic neurites relative to Aβ42 are not reduced by nimodipine treatment. **(**A) Representative images of sections stained with anti-LAMP1 antibody (green) for dystrophic neurites and anti-Aβ42antibody (red) for amyloid plaques. B) Quantification of the ratio of LAMP1:Aβ42. Each plaque was defined as a region of interest, and area covered by LAMP1 and Aβ42 staining measured and used to calculate LAMP1:Aβ42 ratios, then ratios were averaged for plaques in the cortex or hippocampus. There were no significant differences between vehicle or nimodipine-treated mice. C) When plaques were stratified by area of Aβ42 staining, we found a clear correspondence between the size of the Aβ42 region and the ratio of LAMP1:Aβ42, with smaller plaques having higher ratios, indicating a proportionally greater amounts of dystrophic neurites. The decrease in LAMP1:Aβ42 ratio with increasing Aβ42 area was similar between vehicle and nimodipine-treated mice, with no significant differences between the two groups for any size range of plaques. (D) While the number of plaques per mouse varies, the distribution of plaques over the size range is very consistent between mice and is not affected by nimodipine treatment.

Since bulk measures of protein accumulation in dystrophic neurites by western blot may miss small differences, we used immunofluorescence microscopy to determine the amount of LAMP1 positive area relative to Aβ42 area for each plaque (Fig 5). This method may be able to detect smaller differences in the relative amount of neuritic dystrophy per plaque. Using NIS Elements software, each plaque was defined as region of interest (ROI), then the ratio of pixels positive for LAMP1 to pixels positive for Aβ42 was determined. When we averaged the ratio of LAMP1:Aβ42 for total plaques of each mouse, we found no difference between vehicle and nimodipine-treated mice (Fig 5B). Since a previous study indicated that smaller, actively growing plaques have a proportionally larger halo of dystrophic neurites [43], we stratified the plaques based on Aβ42-positive area to determine whether specific size ranges of plaques were affected by nimodipine treatment. When we stratified plaques by area, a significant difference in ratio associated with size became apparent, with smaller plaques having proportionally more LAMP1 in both hippocampus and cortex (Fig 5C). However, we found no difference between vehicle and nimodipine-treated mice for any size plaque, indicating that there was no effect of nimodipine on neuritic dystrophy.

**Fig 6:**
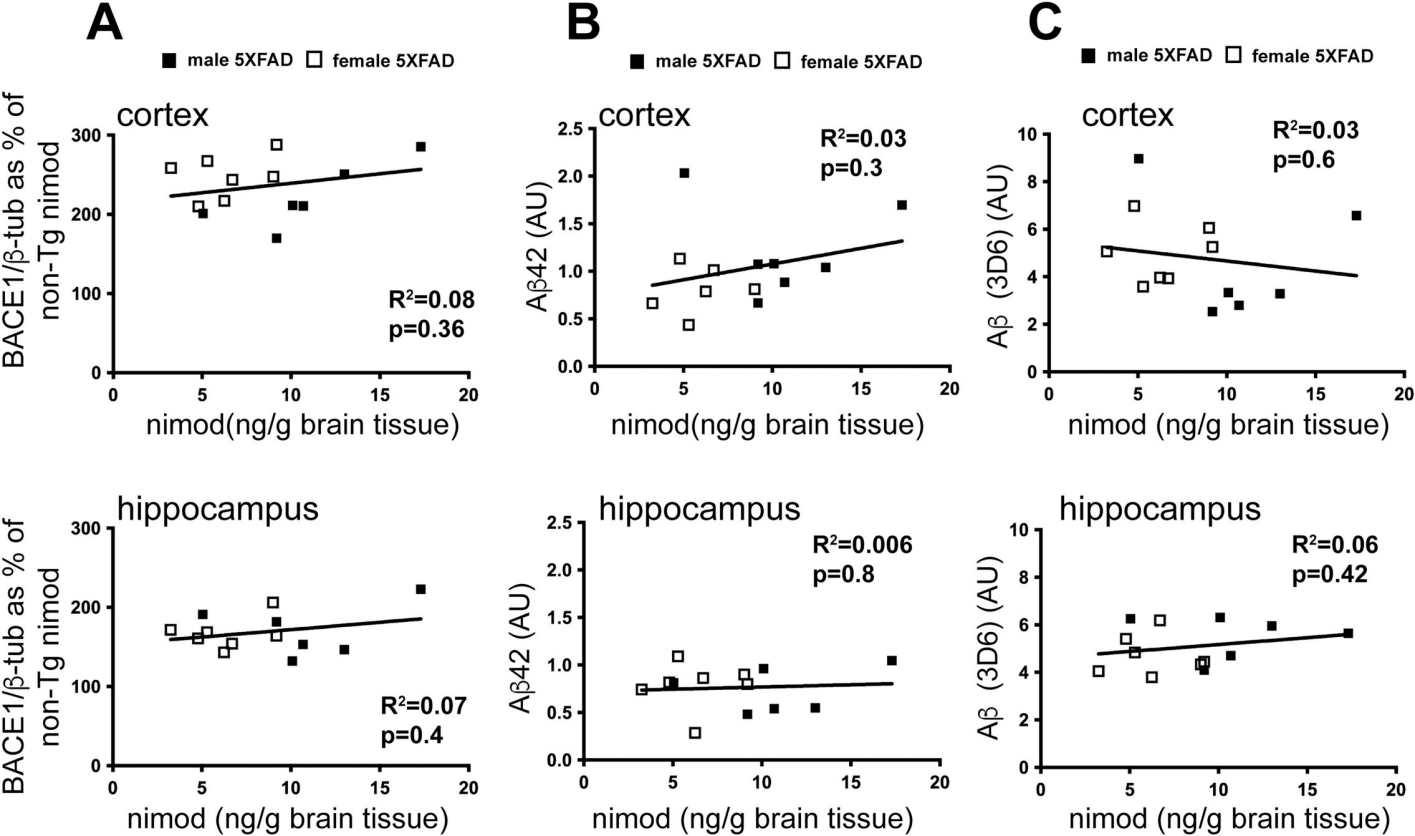
BACE1 and Aβ levels are not correlated with nimodipine levels in 5XFAD transgenic mice. **(**A) BACE1 as measured by western blot (shown in Fig 2), does not correlate with nimodipine levels as measured by mass spectrometry in either cortex or hippocampus. (B) There is also no correlation between Aβ42 levels and nimodipine concentration in the brain or (C) between Aβ and nimodipine.

Since we saw high variation in the concentrations of nimodipine in the brains of treated mice, we correlated nimodipine levels with BACE1 and Aβ immunosignals to determine if the highest concentrations of nimodipine were associated with lower BACE1 and Aβ accumulation. We found no significant correlation between nimodipine levels and BACE1 protein or Aβ levels in the 5XFAD transgenic mice, suggesting nimodipine has no influence on amyloid pathology (Fig 6).

## Discussion

In this study, we analyzed various aspects of amyloid pathology in the 5XFAD amyloidosis model after 6 months of oral nimodipine treatment. We found that while nimodipine did penetrate into the brain, and was well-tolerated, there were no effects on amyloid deposition or the amount of neuritic dystrophy that formed around plaques. Immunoblot analyses of proteins associated with neuritic dystrophy, (BACE1, LAMP1, and the LC3B II/I ratio) showed no decrease in neuritic dystrophy, nor did image analysis quantifying the amount of LAMP1 relative to Aβ42 plaque size. We also quantified amyloid pathology by biochemical and histological methods and found no effect of nimodipine on these measures. There were no significant effects on behavior or blood pressure either, raising the concern that the amount of nimodipine given was too low to affect calcium levels in neurons. Given the difficulties of measuring resting state calcium levels in vivo, we did not perform experiments to definitively quantify the effects of nimodipine on resting calcium in neurons and plaque associated dystrophic neurites. Most genetically encoded chemical calcium sensors are best suited for measuring change in calcium level over time, not to comparing steady-state levels of calcium in different neurons, or even different animals, due to difficulty of normalizing for calcium sensor expression or loading. However, calcium imaging should be pursued to deepen insight into the effects of nimodipine in the context of amyloid pathology.

Despite the lack of data directly measuring the calcium levels, based on information in the literature, we surmise that the amount of nimodipine administered was sufficient to achieve L-type calcium channel inhibition in the brain. Based on the weight of food consumed per cage and the average weight of the mice, females received doses ranging from 40–51 mg/kg/day, while males received 33–41 mg/kg/day. In comparison, oral nimodipine treatment equivalent to 4.8 mg/kg/day in a 75kg human improves outcome after subarachnoid hemorrhage [44]. Mice treated with 4 or 8 mg/kg/day nimodipine during recovery from induced subarachnoid hemorrhage show improved cognitive function compared to vehicle treated animals [45]. In addition, other studies using a dosing regime of nimodipine chow similar to ours reported biological effects such as resistance to methylmercury neurotoxicity [46,47]. It is possible that higher brain concentrations of nimodipine are needed to reduce Aβ-associated increases in calcium levels of neurons that give rise to dystrophic neurites. Achieving higher brain nimodipine levels would be possible using intravenous injection, but this approach is less conducive to long term dosing and could result in larger drug concentration fluctuations over a 24 hour period. Overall, these results suggest that the calcium dysregulation associated with AD is mediated by another type of calcium channel and targeting these channels would be more effective.

It is also possible that nimodipine treatment might have had a significant effect during an even earlier phase of plaque growth. However, this scenario is unlikely since treatment was started at the beginning of plaque formation in the 5XFAD model [31]. Moreover, preliminary data from a smaller cohort of mice harvested at 5 months of age (after three months of nimodipine treatment) did not show any differences in levels of amyloid pathology, BACE1 or other dystrophic neurite markers by immunoblotting or immunofluorescence (not shown). Recently, it was shown that nimodipine blocks Aβ42-induced mitochondrial toxicity in microglia, likely through an off-target effect, and prevents proinflammatory effects of Aβ [48]. It is possible that there are effects on microglial or astrocytic reaction to amyloid plaques in response to nimodipine treatment; this may be pursued in future work.

There is also evidence that other calcium channel drugs could be used to ameliorate AD pathology. Other DHPs, such as nitrendipine and nilvadipine, have shown positive effects in delaying dementia onset [22] and slowing cognitive decline in people with mild cognitive impairment [49]. Verapamil, a phenylalkylamine class calcium channel blocker, has wide ranging cellular effects and improves cognition in rodent models, but its effects in AD are unclear (reviewed in [50]).

One unexpected result of this study was that we could not confirm the cognitive impairment in 5XFAD compared to non-Tg mice in Y-maze or fear conditioning tests. Both non-Tg and 5XFAD mice alternated greater than 50% of the time in Y-maze and increased freezing in response to tone and context in fear conditioning, indicating normal learning and memory in both groups. The initial characterizations of the 5XFAD line [31,51] and subsequent studies [52] have showed learning and memory deficits in 5XFAD mice after 4–6 months of age. Differences between the trace fear conditioning paradigm used in the original characterization [51] and our study may be responsible for the discrepancy in results, at least in part (see “S_9_Supplemental methods” for details). However, the Y-maze protocols used in the two studies were identical and thus cannot explain the discrepancy. We speculate that the most likely cause of the discrepant behavioral results may be the consequence of genetic drift of our 5XFAD colony since its original behavioral characterization in 2007, as we recently reported no difference in performance on these tests in another cohort of 5 month old 5XFAD and non-Tg littermates [42]. Of note, it is known that modifier genes measurably influence the degree of cognitive impairment caused by the 5XFAD transgenes [52].

Importantly, while this study did not indicate any benefit of chronic nimodipine treatment for amyloid pathology in mice, it has demonstrated that chronic nimodipine exposure does not increase the generation or deposition of amyloid. This fact is relevant for clinical studies on the use of nimodipine to restore progranulin expression in progranulin mutation carriers who will develop frontotemporal lobe dementia [53], which could be worsened by increased amyloid deposition. Nimodipine has also shown preclinical promise in treatment of spinal cord injuries and experimental autoimmune encephalomyelitis, an animal model of multiple sclerosis [54,55], and could potentially move into clinical trials for these indications [56].

## Supporting information

S1 Dataset (XLSX)

S2 Dataset (XLSX)

S3 Dataset (XLSX)

S4 Dataset (XLSX)

S5 Dataset (XLSX)

S6 Dataset (XLSX)

S1 Fig No behavioral differences detected between 5XFAD and non-Tg littermates treated with either vehicle or nimodipine chow. (A-C) At 7.5 months of age, two weeks before sacrifice, 5XFAD and non-transgenic mice treated with either vehicle or nimodipine were subject to behavioral analysis in Y maze and fear conditioning tests. (A) No significant difference in memory as measured by Y maze was observed between the groups. (B) In context-based fear conditioning there was also no significant difference between the groups. (C) In cue-based fear conditioning, all groups except nimodipine treated 5XFAD showed significantly more freezing during the cued tone than before, indicating they had learned to associate the tone with foot shock. There was no difference between the groups in the amount of freezing during the tone. The only significant difference was in the amount of freezing in the time before the tone, with the nimodipine treated 5XFAD group freezing more.
(PDF)

S1 Raw images (PDF)

S1 File (DOCX)

## References

[1] Elmaleh DR, Farlow MR, Conti PS, Tompkins RG, Kundakovic L, Tanzi RE. Developing Effective Alzheimer’s Disease Therapies: Clinical Experience and Future Directions. J Alzheimers Dis. 2019;71(3):715–32. doi: 10.3233/JAD-190507 31476157 PMC6839593

[2] Long JM, Holtzman DM. Alzheimer Disease: An Update on Pathobiology and Treatment Strategies. Cell. 2019;179(2):312–39. doi: 10.1016/j.cell.2019.09.001 31564456 PMC6778042

[3] Hussain I, Powell D, Howlett DR, Tew DG, Meek TD, Chapman C, et al. Identification of a novel aspartic protease (Asp 2) as beta-secretase. Molecular and cellular neurosciences. 1999;14(6):419–27. doi: 10.1006/mcne.1999.0811 10656250

[4] Sinha S, Anderson JP, Barbour R, Basi GS, Caccavello R, Davis D, et al. Purification and cloning of amyloid precursor protein beta-secretase from human brain. Nature. 1999;402(6761):537–40. doi: 10.1038/990114 10591214

[5] Vassar R, Bennett BD, Babu-Khan S, Kahn S, Mendiaz EA, Denis P, et al. Beta-Secretase cleavage of Alzheimer’s amyloid precursor protein by the transmembrane aspartic protease BACE. Science. 1999;286(5440):735–41. doi: 10.1126/science.286.5440.735 10531052

[6] Yan R, Bienkowski MJ, Shuck ME, Miao H, Tory MC, Pauley AM, et al. Membrane-anchored aspartyl protease with Alzheimer’s disease beta-secretase activity. Nature. 1999;402(6761):533–7. doi: 10.1038/990107 10591213

[7] Selkoe DJ, Hardy J. The amyloid hypothesis of Alzheimer’s disease at 25 years. EMBO Mol Med. 2016;8(6):595–608. doi: 10.15252/emmm.201606210 27025652 PMC4888851

[8] Khachaturian ZS. Towards theories of brain aging. In: Kay DS, editor. Handbook of studies on psychiatry and old age. Amsterdam: Elsevier Science Publishers, B. V.; 1984. p. 7–30.

[9] Alzheimer’s Association Calcium Hypothesis W. Calcium Hypothesis of Alzheimer’s disease and brain aging: A framework for integrating new evidence into a comprehensive theory of pathogenesis. Alzheimers Dement. 2017;13(2):178–82 e17. doi: 10.1016/j.jalz.2016.12.006 28061328

[10] Demuro A, Mina E, Kayed R, Milton SC, Parker I, Glabe CG. Calcium dysregulation and membrane disruption as a ubiquitous neurotoxic mechanism of soluble amyloid oligomers. J Biol Chem. 2005;280(17):17294–300. doi: 10.1074/jbc.M500997200 15722360

[11] Alberdi E, Sanchez-Gomez MV, Cavaliere F, Perez-Samartin A, Zugaza JL, Trullas R, et al. Amyloid beta oligomers induce Ca2+ dysregulation and neuronal death through activation of ionotropic glutamate receptors. Cell Calcium. 2010;47(3):264–72. doi: 10.1016/j.ceca.2009.12.010 20061018

[12] Nimmrich V, Grimm C, Draguhn A, Barghorn S, Lehmann A, Schoemaker H, et al. Amyloid beta oligomers (A beta(1–42) globulomer) suppress spontaneous synaptic activity by inhibition of P/Q-type calcium currents. J Neurosci. 2008;28(4):788–97. doi: 10.1523/JNEUROSCI.4771-07.2008 18216187 PMC6671006

[13] Busche MA, Chen X, Henning HA, Reichwald J, Staufenbiel M, Sakmann B, et al. Critical role of soluble amyloid-beta for early hippocampal hyperactivity in a mouse model of Alzheimer’s disease. Proceedings of the National Academy of Sciences of the United States of America. 2012;109(22):8740–5. doi: 10.1073/pnas.1206171109 22592800 PMC3365221

[14] Busche MA, Eichhoff G, Adelsberger H, Abramowski D, Wiederhold KH, Haass C, et al. Clusters of hyperactive neurons near amyloid plaques in a mouse model of Alzheimer’s disease. Science. 2008;321(5896):1686–9. doi: 10.1126/science.1162844 18802001

[15] Kuchibhotla KV, Goldman ST, Lattarulo CR, Wu HY, Hyman BT, Bacskai BJ. Abeta plaques lead to aberrant regulation of calcium homeostasis in vivo resulting in structural and functional disruption of neuronal networks. Neuron. 2008;59(2):214–25. doi: 10.1016/j.neuron.2008.06.008 18667150 PMC2578820

[16] Xie H, Guan J, Borrelli LA, Xu J, Serrano-Pozo A, Bacskai BJ. Mitochondrial alterations near amyloid plaques in an Alzheimer’s disease mouse model. J Neurosci. 2013;33(43):17042–51. doi: 10.1523/JNEUROSCI.1836-13.2013 24155308 PMC3807029

[17] Sadleir KR, Kandalepas PC, Buggia-Prevot V, Nicholson DA, Thinakaran G, Vassar R. Presynaptic dystrophic neurites surrounding amyloid plaques are sites of microtubule disruption, BACE1 elevation, and increased Abeta generation in Alzheimer’s disease. Acta Neuropathol. 2016;132(2):235–56. doi: 10.1007/s00401-016-1558-9 26993139 PMC4947125

[18] Lerdkrai C, Asavapanumas N, Brawek B, Kovalchuk Y, Mojtahedi N, Olmedillas Del Moral M, et al. Intracellular Ca(2+) stores control in vivo neuronal hyperactivity in a mouse model of Alzheimer’s disease. Proceedings of the National Academy of Sciences of the United States of America. 2018;115(6):E1279–E88. doi: 10.1073/pnas.1714409115 29358403 PMC5819404

[19] Zhang H, Sun S, Herreman A, De Strooper B, Bezprozvanny I. Role of presenilins in neuronal calcium homeostasis. J Neurosci. 2010;30(25):8566–80. doi: 10.1523/JNEUROSCI.1554-10.2010 20573903 PMC2906098

[20] Fritze J, Walden J. Clinical findings with nimodipine in dementia: test of the calcium hypothesis. J Neural Transm Suppl. 1995;46:439–53. 8821080

[21] Tollefson GD. Short-term effects of the calcium channel blocker nimodipine (Bay-e-9736) in the management of primary degenerative dementia. Biol Psychiatry. 1990;27(10):1133–42. doi: 10.1016/0006-3223(90)90050-c 2187540

[22] Forette F, Seux ML, Staessen JA, Thijs L, Birkenhager WH, Babarskiene MR, et al. Prevention of dementia in randomised double-blind placebo-controlled Systolic Hypertension in Europe (Syst-Eur) trial. Lancet. 1998;352(9137):1347–51. doi: 10.1016/s0140-6736(98)03086-4 9802273

[23] Cohen MW, Jones OT, Angelides KJ. Distribution of Ca2+ channels on frog motor nerve terminals revealed by fluorescent omega-conotoxin. J Neurosci. 1991;11(4):1032–9. doi: 10.1523/JNEUROSCI.11-04-01032.1991 1707093 PMC6575372

[24] Hell JW, Westenbroek RE, Warner C, Ahlijanian MK, Prystay W, Gilbert MM, et al. Identification and differential subcellular localization of the neuronal class C and class D L-type calcium channel alpha 1 subunits. J Cell Biol. 1993;123(4):949–62. doi: 10.1083/jcb.123.4.949 8227151 PMC2200142

[25] Westenbroek RE, Ahlijanian MK, Catterall WA. Clustering of L-type Ca2+ channels at the base of major dendrites in hippocampal pyramidal neurons. Nature. 1990;347(6290):281–4. doi: 10.1038/347281a0 2169591

[26] Larkin JG, Thompson GG, Scobie G, Forrest G, Drennan JE, Brodie MJ. Dihydropyridine calcium antagonists in mice: blood and brain pharmacokinetics and efficacy against pentylenetetrazol seizures. Epilepsia. 1992;33(4):760–9. doi: 10.1111/j.1528-1157.1992.tb02358.x 1628595

[27] Tomassoni D, Lanari A, Silvestrelli G, Traini E, Amenta F. Nimodipine and its use in cerebrovascular disease: evidence from recent preclinical and controlled clinical studies. Clin Exp Hypertens. 2008;30(8):744–66. doi: 10.1080/10641960802580232 19021025

[28] Morich F BF, Lewis JM, Kaiser L, Cutler NR, Escobar JI, Willmer J, et al. Nimodipine in the Treatment of Probable Alzheimer’s Disease: Results of Two Multicentre Trials. Clinical Drug Investigation. 1996;11(4):185–95.

[29] Lopez-Arrieta JM, Birks J. Nimodipine for primary degenerative, mixed and vascular dementia. Cochrane Database Syst Rev. 2002(3):CD000147. doi: 10.1002/14651858.CD000147 12137606

[30] Ishii M, Hiller AJ, Pham L, McGuire MJ, Iadecola C, Wang G. Amyloid-Beta Modulates Low-Threshold Activated Voltage-Gated L-Type Calcium Channels of Arcuate Neuropeptide Y Neurons Leading to Calcium Dysregulation and Hypothalamic Dysfunction. J Neurosci. 2019;39(44):8816–25. doi: 10.1523/JNEUROSCI.0617-19.2019 31537707 PMC6820205

[31] Oakley H, Cole SL, Logan S, Maus E, Shao P, Craft J, et al. Intraneuronal beta-amyloid aggregates, neurodegeneration, and neuron loss in transgenic mice with five familial Alzheimer’s disease mutations: potential factors in amyloid plaque formation. J Neurosci. 2006;26(40):10129–40. doi: 10.1523/JNEUROSCI.1202-06.2006 17021169 PMC6674618

[32] Zhao J, Fu Y, Yasvoina M, Shao P, Hitt B, O’Connor T, et al. Beta-site amyloid precursor protein cleaving enzyme 1 levels become elevated in neurons around amyloid plaques: implications for Alzheimer’s disease pathogenesis. J Neurosci. 2007;27(14):3639–49. doi: 10.1523/JNEUROSCI.4396-06.2007 17409228 PMC6672403

[33] Langley MS, Sorkin EM. Nimodipine. A review of its pharmacodynamic and pharmacokinetic properties, and therapeutic potential in cerebrovascular disease. Drugs. 1989;37(5):669–99. doi: 10.2165/00003495-198937050-00004 2663415

[34] Hilliard LM, Sampson AK, Brown RD, Denton KM. The "his and hers" of the renin-angiotensin system. Curr Hypertens Rep. 2013;15(1):71–9. doi: 10.1007/s11906-012-0319-y 23180053

[35] Sandberg K, Ji H. Sex differences in primary hypertension. Biol Sex Differ. 2012;3(1):7. doi: 10.1186/2042-6410-3-7 22417477 PMC3331829

[36] Gowrishankar S, Yuan P, Wu Y, Schrag M, Paradise S, Grutzendler J, et al. Massive accumulation of luminal protease-deficient axonal lysosomes at Alzheimer’s disease amyloid plaques. Proceedings of the National Academy of Sciences of the United States of America. 2015. doi: 10.1073/pnas.1510329112 26124111 PMC4507205

[37] Kandalepas PC, Sadleir KR, Eimer WA, Zhao J, Nicholson DA, Vassar R. The Alzheimer’s beta-secretase BACE1 localizes to normal presynaptic terminals and to dystrophic presynaptic terminals surrounding amyloid plaques. Acta Neuropathol. 2013;126(3):329–52. doi: 10.1007/s00401-013-1152-3 23820808 PMC3753469

[38] Yuan P, Condello C, Keene CD, Wang Y, Bird TD, Paul SM, et al. TREM2 Haplodeficiency in Mice and Humans Impairs the Microglia Barrier Function Leading to Decreased Amyloid Compaction and Severe Axonal Dystrophy. Neuron. 2016;90(4):724–39. doi: 10.1016/j.neuron.2016.05.003 27196974 PMC4898967

[39] Sadleir KR, Eimer WA, Cole SL, Vassar R. Abeta reduction in BACE1 heterozygous null 5XFAD mice is associated with transgenic APP level. Molecular neurodegeneration. 2015;10:1. doi: 10.1186/1750-1326-10-1 25567526 PMC4297413

[40] Sadleir KR, Eimer WA, Kaufman RJ, Osten P, Vassar R. Genetic inhibition of phosphorylation of the translation initiation factor eIF2alpha does not block Abeta-dependent elevation of BACE1 and APP levels or reduce amyloid pathology in a mouse model of Alzheimer’s disease. PLoS One. 2014;9(7):e101643. doi: 10.1371/journal.pone.0101643 24992504 PMC4081565

[41] Rocchi A, Yamamoto S, Ting T, Fan Y, Sadleir K, Wang Y, et al. A Becn1 mutation mediates hyperactive autophagic sequestration of amyloid oligomers and improved cognition in Alzheimer’s disease. PLoS Genet. 2017;13(8):e1006962. doi: 10.1371/journal.pgen.1006962 28806762 PMC5570506

[42] Sadleir KR, Popovoic J, Zhu W, Reidel CT, Do H, Silverman RB, et al. Pregabalin Treatment does not Affect Amyloid Pathology in 5XFAD Mice. Curr Alzheimer Res. 2021;18(4):283–97. doi: 10.2174/1567205018666210713125333 34259145 PMC9527523

[43] Condello C, Schain A, Grutzendler J. Multicolor time-stamp reveals the dynamics and toxicity of amyloid deposition. Sci Rep. 2011;1:19. doi: 10.1038/srep00019 22355538 PMC3216507

[44] Pickard JD, Murray GD, Illingworth R, Shaw MD, Teasdale GM, Foy PM, et al. Effect of oral nimodipine on cerebral infarction and outcome after subarachnoid haemorrhage: British aneurysm nimodipine trial. BMJ. 1989;298(6674):636–42. doi: 10.1136/bmj.298.6674.636 2496789 PMC1835889

[45] Mesis RG, Wang H, Lombard FW, Yates R, Vitek MP, Borel CO, et al. Dissociation between vasospasm and functional improvement in a murine model of subarachnoid hemorrhage. Neurosurg Focus. 2006;21(3):E4. doi: 10.3171/foc.2006.21.3.4 17029343

[46] Bailey JM, Hutsell BA, Newland MC. Dietary nimodipine delays the onset of methylmercury neurotoxicity in mice. Neurotoxicology. 2013;37:108–17. doi: 10.1016/j.neuro.2013.03.011 23583802 PMC3696396

[47] Hoffman DJ, Newland MC. A microstructural analysis distinguishes motor and motivational influences over voluntary running in animals chronically exposed to methylmercury and nimodipine. Neurotoxicology. 2016;54:127–39. doi: 10.1016/j.neuro.2016.04.009 27095634

[48] Chiozzi P, Sarti AC, Sanz JM, Giuliani AL, Adinolfi E, Vultaggio-Poma V, et al. Amyloid beta-dependent mitochondrial toxicity in mouse microglia requires P2X7 receptor expression and is prevented by nimodipine. Sci Rep. 2019;9(1):6475. doi: 10.1038/s41598-019-42931-2 31019207 PMC6482182

[49] Hanyu H, Hirao K, Shimizu S, Sato T, Kiuchi A, Iwamoto T. Nilvadipine prevents cognitive decline of patients with mild cognitive impairment. Int J Geriatr Psychiatry. 2007;22(12):1264–6. doi: 10.1002/gps.1851 18033677

[50] Popovic N, Morales-Delgado N, Vidal Mena D, Alonso A, Pascual Martinez M, Caballero Bleda M, et al. Verapamil and Alzheimer’s Disease: Past, Present, and Future. Front Pharmacol. 2020;11:562. doi: 10.3389/fphar.2020.00562 32431612 PMC7214748

[51] Ohno M, Cole SL, Yasvoina M, Zhao J, Citron M, Berry R, et al. BACE1 gene deletion prevents neuron loss and memory deficits in 5XFAD APP/PS1 transgenic mice. Neurobiol Dis. 2007;26(1):134–45. doi: 10.1016/j.nbd.2006.12.008 17258906 PMC1876698

[52] Neuner SM, Heuer SE, Zhang JG, Philip VM, Kaczorowski CC. Identification of Pre-symptomatic Gene Signatures That Predict Resilience to Cognitive Decline in the Genetically Diverse AD-BXD Model. Front Genet. 2019;10:35. doi: 10.3389/fgene.2019.00035 30787942 PMC6372563

[53] Sha SJ, Miller ZA, Min SW, Zhou Y, Brown J, Mitic LL, et al. An 8-week, open-label, dose-finding study of nimodipine for the treatment of progranulin insufficiency from GRN gene mutations. Alzheimers Dement (N Y). 2017;3(4):507–12. doi: 10.1016/j.trci.2017.08.002 29124108 PMC5671622

[54] Marcantoni M, Fuchs A, Low P, Bartsch D, Kiehn O, Bellardita C. Early delivery and prolonged treatment with nimodipine prevents the development of spasticity after spinal cord injury in mice. Sci Transl Med. 2020;12(539). doi: 10.1126/scitranslmed.aay0167 32295897

[55] Schampel A, Volovitch O, Koeniger T, Scholz CJ, Jorg S, Linker RA, et al. Nimodipine fosters remyelination in a mouse model of multiple sclerosis and induces microglia-specific apoptosis. Proceedings of the National Academy of Sciences of the United States of America. 2017;114(16):E3295–E304. doi: 10.1073/pnas.1620052114 28381594 PMC5402421

[56] Gottle P, Forster M, Weyers V, Kury P, Rejdak K, Hartung HP, et al. An unmet clinical need: roads to remyelination in MS. Neurol Res Pract. 2019;1:21. doi: 10.1186/s42466-019-0026-0 33324887 PMC7650135

