# Supplementary material for: Oral nimodipine treatment has no effect on amyloid pathology or neuritic dystrophy in the 5XFAD mouse model of amyloidosis
Source: PLoS One. 2022 Feb 2;17(2):e0263332. doi: 10.1371/journal.pone.0263332 (PMC8809624; doi:10.1371/journal.pone.0263332)
Supplement: S1 Fig — (A-C) At 7.5 months of age, two weeks before sacrifice, 5XFAD and non-transgenic mice treated with either vehicle or nimodipine were subject to behavioral analysis in Y maze and fear conditioning tests. (A) No significant difference in memory as measured by Y maze was observed between the groups. (B) In context-based fear conditioning there was also no significant difference between the groups. (C) In cue-based fear conditioning, all groups except nimodipine treated 5XFAD showed significantly more freezing during the cued tone than before, indicating they had learned to associate the tone with foot shock. There was no difference between the groups in the amount of freezing during the tone. The only significant difference was in the amount of freezing in the time before the tone, with the nimodipine treated 5XFAD group freezing more. (PDF) [file pone.0263332.s007.pdf]

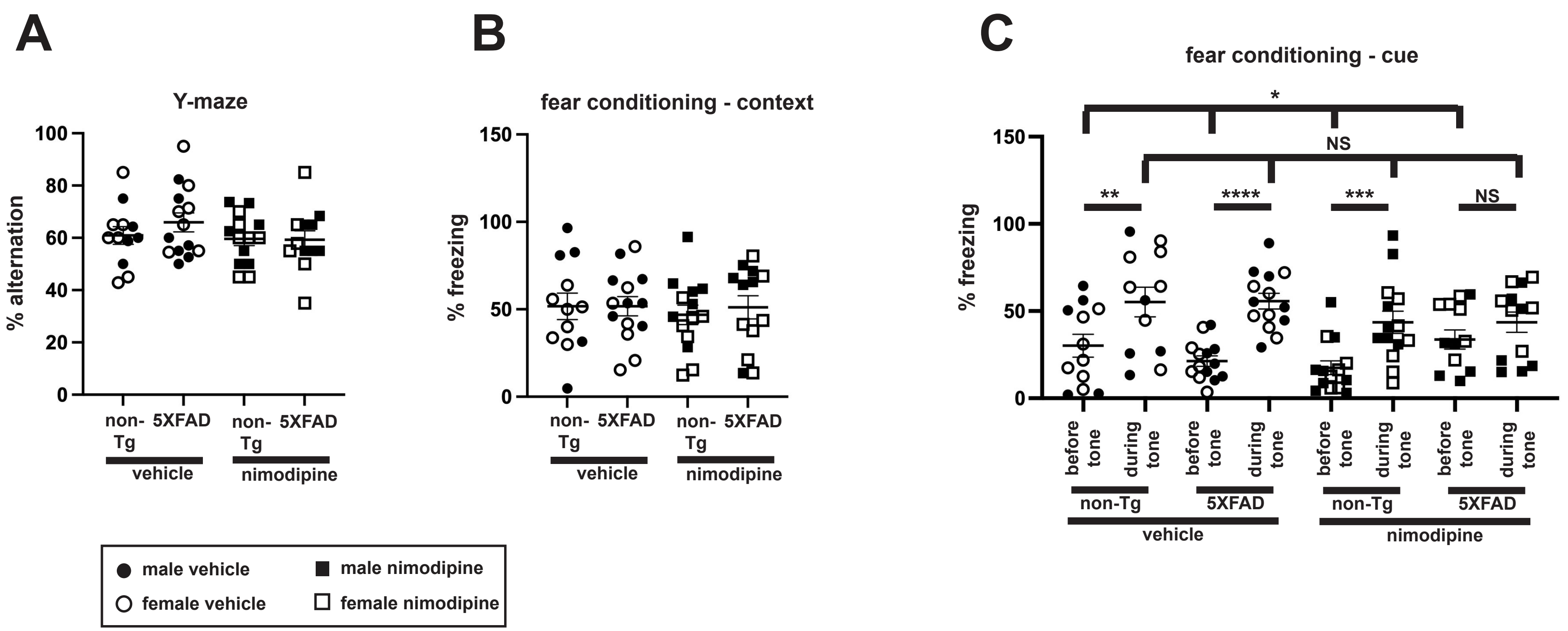

Supplemental Figure 1: No behavioral differences detected between 5XFAD and non-Tg littermates treated with either vehicle or nimodipine chow. (A-C) At 7.5 months of age, two weeks before sacrifice, 5XFAD and non-transgenic mice treated with either vehicle or nimodipine were subject to behavioral analysis in Y maze and fear conditioning tests. (A) No significant difference in memory as measured by Y maze was observed between the groups. (B) In context based fear conditioning there was also no significant difference between the groups. (C) In cue-based fear conditioning, all groups except nimodipine treated 5XFAD showed significantly more freezing during the cued tone than before, indicating they had learned to associate the tone with foot shock. There was no difference between the groups in the amount of freezing during the tone. The only significant difference was in the amount of freezing in the time before the tone, with the nimodipine treated 5XFAD group freezing more.

Supp. Fig. 1
