# Supplementary material for: Oral nimodipine treatment has no effect on amyloid pathology or neuritic dystrophy in the 5XFAD mouse model of amyloidosis
Source: PLoS One. 2022 Feb 2;17(2):e0263332. doi: 10.1371/journal.pone.0263332 (PMC8809624; doi:10.1371/journal.pone.0263332)

Figure 2, Panel A

m Tg- Veh
M Tg- nim
M Tg+ veh
M Tg+ nim
F Tg- veh
F Tg- nim
F Tg+ veh
F Tg+ nim

——— indicates lanes shown in Figure 2, panel A

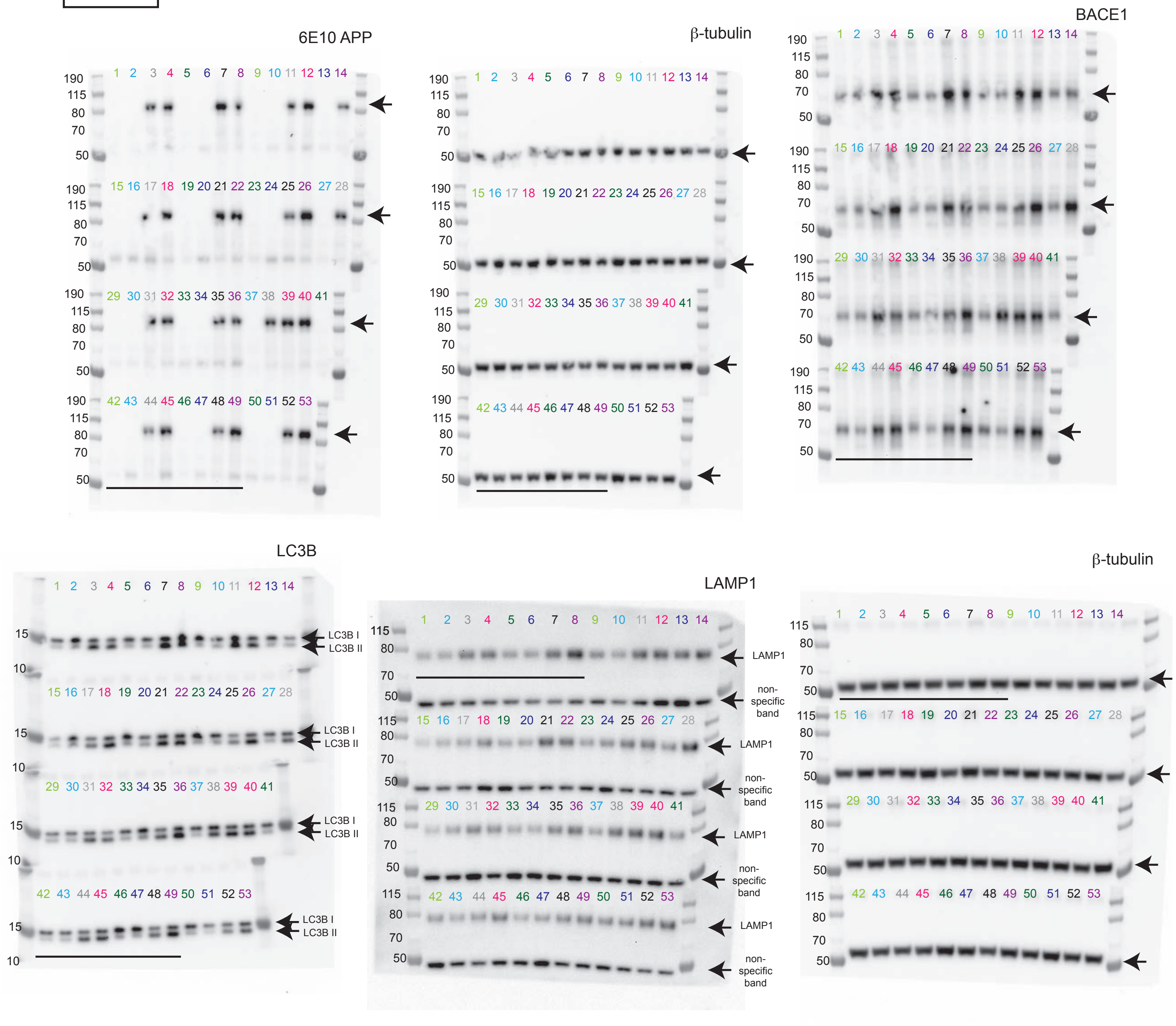

Figure 2, Panel F

m Tg- Veh
M Tg- nim
M Tg+ veh
M Tg+ nim
F Tg- veh
F Tg- nim
F Tg+ veh
F Tg+ nim

——— indicates lanes shown in Figure 2, panel F

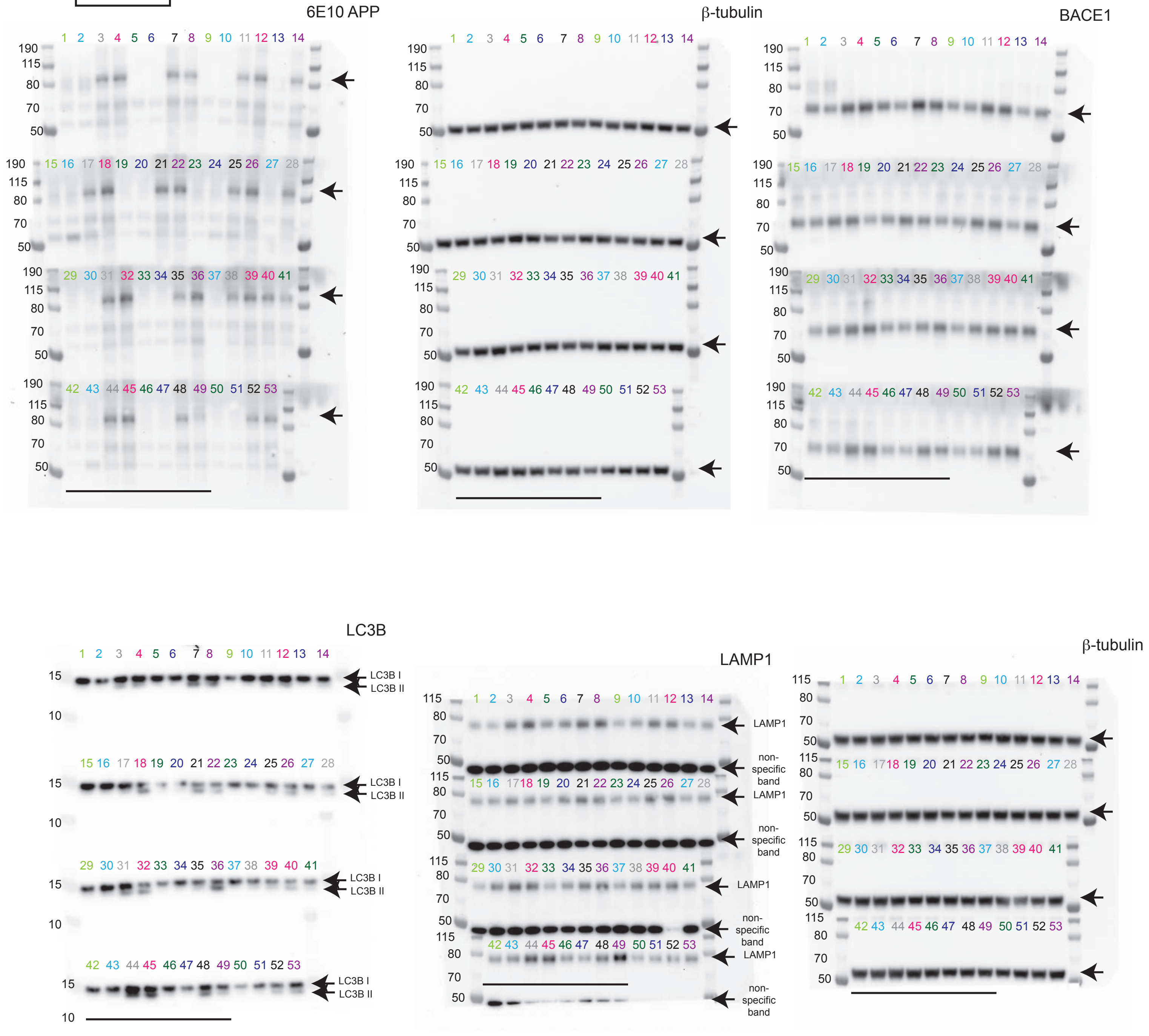

Figure 3, Panel A

m Tg- Veh
M Tg- nim
M Tg+ veh
M Tg+ nim
F Tg- veh
F Tg- nim
F Tg+ veh
F Tg+ nim

☐ indicates region shown in Figure 3

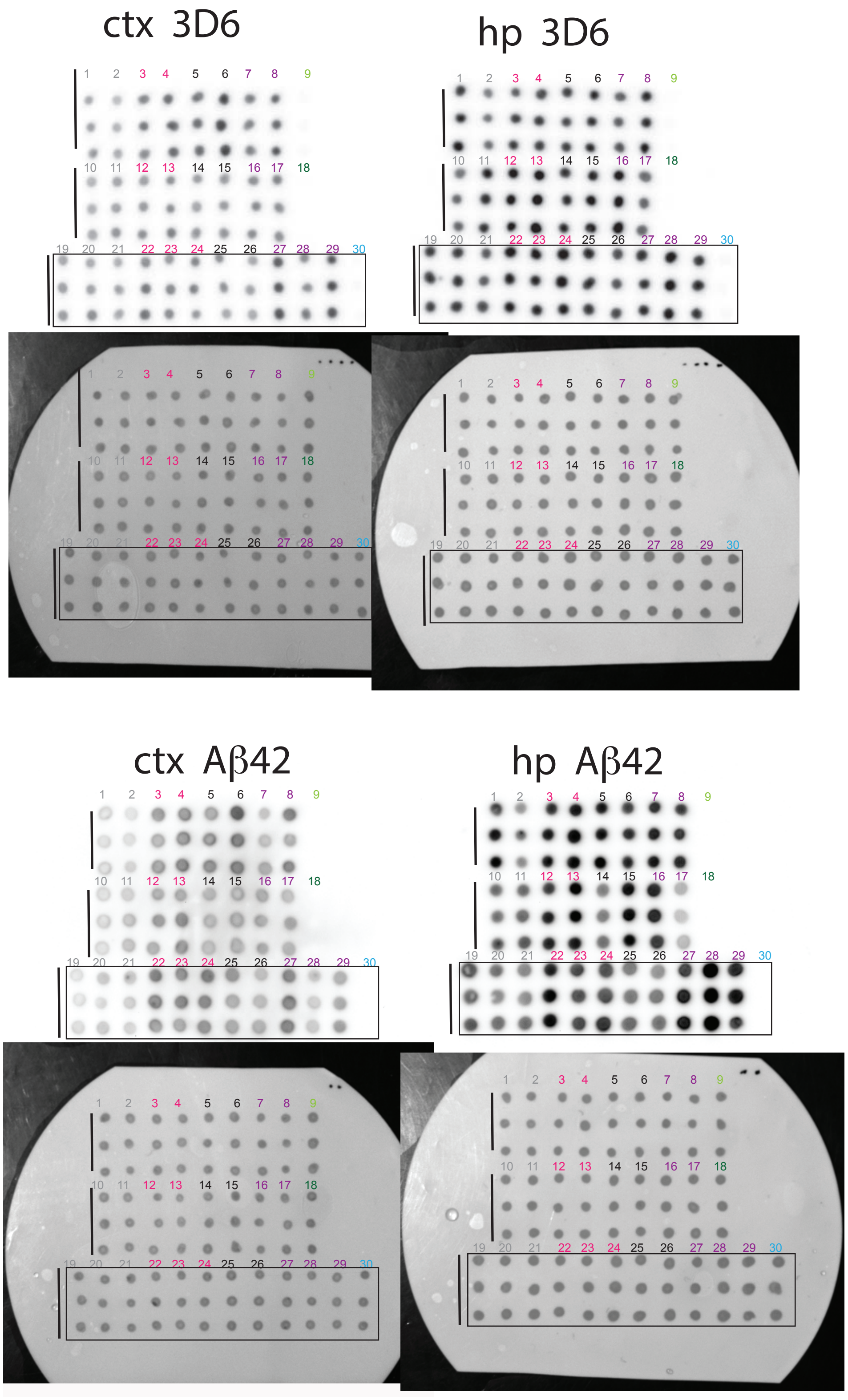

Supplement: S1 Raw images — (PDF) [file pone.0263332.s008.pdf]
