# Supplementary material for: Oral nimodipine treatment has no effect on amyloid pathology or neuritic dystrophy in the 5XFAD mouse model of amyloidosis
Source: PLoS One. 2022 Feb 2;17(2):e0263332. doi: 10.1371/journal.pone.0263332 (PMC8809624; doi:10.1371/journal.pone.0263332)
Supplement: S1 File — (DOCX) [file pone.0263332.s009.docx]

**Supplemental Methods**

**Behavioral testing:**

Fear conditioning:

During Day 1 training mice were placed in the training chamber in which they receive 3 conditioning trials with an intertrial interval of 5 minutes. Each trial consists of a 30 sec tone (approximately 80 db) followed by a brief (0.5 – 1.0 sec) and mild (0.5 -1.0 ma) foot-shock through a grid floor at the bottom of the cage. The shock was delivered after a 20 sec stimulus free interval (trace conditioning). The animal was returned to its home cage after training. FreezeFrame software was used to control the stimuli and to record motion of the animal. The chamber was cleaned with ethanol and Clidox between mice to eliminate any odors. After a delay of 24 hours, the mouse was returned to the same conditioning chamber for 5 min and its movements were recorded with the use of a video camera to test for contextual conditioning. Very low levels of movement in this environment indicated freezing or “fear” that is associated with the context of the conditioning chamber. Approximately 30-60 minutes after the context test the animal was placed in a novel context (no grid floor, new visual cues, new shape, new odor) and tested for cue-signaled fear by presenting the original conditioning tone.

Y maze:

Mice were placed in the “start” arm of the Y-maze, then allowed to explore freely for 5 minutes. A video camera and LimeLight software were used to record the animals’ movements. After five minutes, the animal was returned to its home cage. The maze was cleaned with ethanol and Clidox between mice to eliminate any odors. LimeLight was used to score arm entries and percent alternation. All behavioral testing was performed in Northwestern’s Behavioral Phenotyping Core, by core staff, using animal protocols approved by Northwestern IACUC.
